# Serum Metabonomics Reveals Key Metabolites in Different Types of Childhood Short Stature

**DOI:** 10.3389/fphar.2022.818952

**Published:** 2022-05-05

**Authors:** Guoyou Chen, Jinming Wang, Yisi Jing, Chunxiang Li, Wenyue Zhang, Shuang Yang, Ye Song, Xin Wang, Jincheng Liu, Dejun Yu, Zhichun Xu

**Affiliations:** ^1^ Daqing Campus, Harbin Medical University, Daqing, China; ^2^ Gynecology Department, Dating Oil Field General Hospital, Daqing, China; ^3^ Fifth Affiliated Hospital, Harbin Medical University, Daqing, China

**Keywords:** different types, human serum, metabonomics, key metabolites, childhood short stature (SS)

## Abstract

Nowadays, short stature (SS) in childhood is a common condition encountered by pediatricians, with an increase in not just a few families. Various studies related to the variations in key metabolites and their biological mechanisms that lead to SS have increased our understanding of the pathophysiology of the disease. However, little is known about the role of metabolite variation in different types of childhood SS that influence these biological processes and whether the understanding of the key metabolites from different types of childhood SS would predict the disease progression better. We performed a systematic investigation using the metabonomics method and studied the correlation between the three groups, namely, the control, idiopathic short stature (ISS), and short stature due to growth hormone deficiency (GHD). We observed that three pathways (viz., purine metabolism, sphingolipid signaling pathway, and sphingolipid metabolism) were significantly enriched in childhood SS. Moreover, we reported that two short peptides (Thr Val Leu Thr Ser and Trp Ile Lys) might play a significant role in childhood SS. Various metabolites in different pathways including 9,10-DiHOME, 12-HETE, 12(13)-EpOME, arachidonic acid methyl ester, glycerophospho-N-arachidonoyl ethanolamine, curvulinic acid (2-acetyl-3,5-dihydroxyphenyl acetic acid), nonanoic acid, and N'-(2,4-dimethylphenyl)-N-methylformamidine in human serum were compared between 60 children diagnosed with SS and 30 normal-height children. More investigations in this area may provide insights and enhance the personalized treatment approaches in clinical practice for SS by elucidating pathophysiology mechanisms of experimental verification.

## Graphical Abstract

SS, short stature; SDS, standard deviation scores; Control, normal height, *n*=30; ISS, idiopathic short stature *n*=31; GHD, short stature caused by growth hormone deficiency, *n*=29.
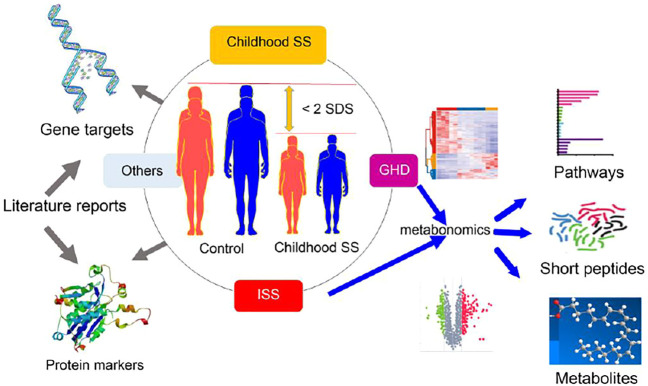

## Introduction

In clinical practice, the therapy of childhood short stature (SS) is often confronted by pediatric endocrinologists and is also an intractable problem (Rogol and Hayden, 2014; Smuel and Yeshayahu, 2017; Ma et al., 2019). Almost half of the pediatric visitors come to consult about short stature (Murray et al., 2018). Childhood short stature, which has various causes, can be categorized under normal and pathological conditions (Ma et al., 2019). The normal condition includes familial short stature and constitutional delay of growth. However, various pathological factors lead to SS, including Turner syndrome, hypothyroidism, chronic diseases, growth hormone deficiency, and idiopathic short stature (Allen and Cuttler, 2013). Short stature during childhood is easily overlooked and embarrassing, which makes an individual vulnerable to psychological disorders, such as low self-esteem, loneliness, academic and job difficulties, and social immaturity (Kim and Park, 2009). Regrettably, many children with SS remain affected by short stature as they grow old (Ma et al., 2019). For example, the probability of developing preterm birth or stillbirth for pregnant women with SS was seen to be relatively higher (Derraik et al., 2016; Ma et al., 2019). Childhood short stature may be more susceptible to chronic diseases such as obesity and insulin resistance. The inactivation of sirtuin 1 (SIRT1) is connected to the progression of insulin resistance associated with SS. Diabetes in people with short stature may be induced later in life with relevance to sirtuin 1 repression (Martins, 2016; Martins, 2017). Therefore, it becomes necessary and urgent to identify critical targets and mechanisms for childhood short stature.

It has been observed that BMI and growth hormone (GH) were negatively correlated with childhood short stature (Bosy-Westphal et al., 2009; Zhao et al., 2021). SIRT1 is a nicotinamide adenine dinucleotide (NAD)-dependent histone deacetylase that is activated in response to calorie restriction (CR) (Yamamoto and Takahashi, 2018). Further investigation between sirtuins, metabolism, and age-associated diseases has implicated the essential role of activation of SIRT1 (Satoh et al., 2011). SIRT1 regulated the adaptive response of the GH–insulin-like growth factor 1 (IGF-1) axis under particular conditions in the liver (Yamamoto et al., 2013). Therefore, the role of SIRT1 activators may be of critical importance in molecular metabolic processes and the treatment of childhood short stature.

Recent studies have identified some possible protein markers in childhood short stature: bone alkaline phosphatase, collagen markers, apolipoprotein (Apo) A-II, Apo C-I, Apo A-II, serum amyloid A4 (SAA4), and transthyretin (TTR) (Crofton et al., 1996; Hellgren et al., 2008; Decker et al., 2013). The most common cause of monogenic short stature is the deficiency of the short-stature homeobox-containing (SHOX) gene (Marchini et al., 2016; Ponomarenko et al., 2020). A study revealed newer mechanistic insights that identified c.1675G > A mutation in receptor tyrosine kinase-like orphan receptor 2 (ROR2) in patients with short stature (Gui et al., 2021). To date, key molecular mechanisms underlying stunted growth and childhood short stature remain equivocal. Meanwhile, a number of children are troubled with SS; it is believed that the knowledge of crucial metabolites will help in rapid diagnosis. Moreover, physiological and pathological mechanisms are also growing significantly and are reliable. Metabonomics is a powerful biological tool commonly used in disease phenotypic studies, which plays a vital role in several aspects such as biomarker discovery, the origin and development of a disease, and the personalized treatment (Nicholson et al., 2005; Huang et al., 2013; Wishart, 2016).

In our study, the serum metabolic profiling of 60 children with SS and 30 normal-height children was investigated using UHPLC-Q-TOF-MS. We explored 10 significant metabolites that are found in human serum in the different types of childhood SS groups (ISS and GHD) compared to the control children group. They were short peptides (Thr Val Leu Thr Ser and Trp Ile Lys and), 9,10-DiHOME, 12-HETE, 12(13)-EpOME, arachidonic acid methyl ester, glycerophospho-N-arachidonoyl ethanolamine, curvulinic acid (2-acetyl-3,5-dihydroxyphenyl acetic acid), nonanoic acid, and N'-(2,4-dimethylphenyl)-N-methylformamidine. These metabolites regulate various pathways, including arachidonic acid metabolism, short peptides metabolism, purine metabolism, sphingolipid signaling pathway, and sphingolipid metabolism in children’s body. Our results explore new therapeutic target metabolites based on metabonomics analysis. The key metabolites discovered will be beneficial in rapid clinical diagnosis and individualized treatment of patients with different types of childhood SS.

## Materials and Methods

### Sample Collection of Children With Short Stature

All patients were recruited from the Fifth Affiliated Hospital of Harbin Medical University. The Ethics Committee approved the study of the Fifth Affiliated Hospital of Harbin Medical University (KY2018003). In addition, the study to determine the childhood short stature was diagnosed according to the 2015 edition of Chinese guidelines for childhood short stature prevention and treatment. On the basis of the cause of SS, the childhood short stature groups were strictly divided into two. The two groups that are defined as ISS (31 SSs) and GHD (29 SSs) comprise the short stature children due to idiopathy or the deficiency of growth hormone, respectively. The controls (30 children) were from physical examination screening. Moreover, the serum for non-targeted metabonomics analysis was collected from 60 SS children and 30 controls from June 2018 to September 2019. Sample information is summed up in Table 1.

**TABLE 1:** Personal basic information.

	Control	ISS	GHD	*P* (ISS *vs*. control)	*P* (GHD *vs.* control)	*P* (ISS *vs.* GHD)
Number	30	31	29			
Gender	16 boys	18 boys	20 boys			
	14 girls	13 girls	9 girls			
Age (years)	8.5 ± 1.3	9.2 ± 2.5	7.9 ± 3.1	0.225	0.098	0.12
Height (cm)	133.4 ± 7.7	119.7 ± 13.0	113 ± 15.1	<0.001	<0.001	0.069
Body weight (kg)	29.5 ± 4.5	30.9 ± 12.5	28.9 ± 17.2	0.572	0.852	0.609
BMI (kg/m2)	16.5 ± 0.9	21 ± 5.9	20.9 ± 6.7	<0.001	<0.001	0.978

Control, normal height; *n* = 30; ISS, idiopathic short stature, *n* = 31; GHD, short stature caused by growth hormone deficiency, *n* = 29; age (years), height (cm), body weight (kg), BMI (kg/m2): mean ± SD, two-tailed *t* test.

### Non-Targeted Metabonomics Analysis of SSs

The chromatographic separation was performed by ultrahigh-pressure liquid chromatography (UHPLC) (Agilent 1290, United States). For purification, the chromatographic column used was ACQUITY UPLC HSS T3 1.8 μm 2.1 × 100 mm (Waters). The mobile phase included 0.1% formic acid in water (part A) and 0.1% formic acid in acetonitrile (part B) (Zhao et al., 2014). The gradient elution was 5% B kept for 1 min, changed linearly to 10% B within 1 min, then changed linearly to 95% B within 12 min, held for 2 min, finally changed linearly to 5% B within 1 min, and held for 3 min. The analytical column and autosampler temperatures were 35 and 4°C, respectively. The sample volume was 5 μL for each run. The column eluent was directly analyzed from the MS system (Zhao et al., 2014).

In total, 200 µL of serum with four volumes of methanol/acetonitrile (1:1, v/v) was extracted. All the samples were shaken for 30 s and subjected to ultrasound for 10 min. Next, the mixture was incubated at −20°C for 2 h to facilitate the precipitation of protein. The serum supernatant was collected after 15 min of centrifugation at 13,000 *g* and dried under vacuum and 4°C and centrifuged before the MS test. The aliquots were reconstituted in 200 μL of acetonitrile/water (1:1) and were shaken for 30 s. Then each sample supernatant after 15-min centrifugation at 13,000 *g* was collected at a volume of 150 µL and analyzed by a UHPLC system (Agilent 1290, United States) coupled with a Q-TOF system (Agilent 6545, United States). The remaining sample supernatants were mixed to make many quality control (QC) samples. The QC samples were carried out after every 15 serum samples.

Data were obtained with auto MS/MS mode from m/z 50-1100. Collision energies for collision-induced dissociation were 20 and 40 V. MS parameters were set as follows: ion source dry gas temperature was at 320°C, N_2_ gas flow was 8 L/min, sheath gas temperature was 350°C, sheath gas flow was 12 L/min, and ion spray voltage was 4000 V (positive ion) and 3500 V (negative ion), respectively.

### Data Collection and Analysis

Data files from the Q-TOF-MS system were converted to the .abf format using the Analysis Base File Converter software. Peak detection, chromatogram deconvolution, and other data processing used MSDIAL3.82 software and aligned with the following parameters: alignment-MS1, tolerance −0.01Da, retention time tolerance-0.2 min, identification accurate mass tolerance (MS1)-0.005Da, (MS2)-0.05Da, and identification score cutoff −60%. For the identification of key metabolites and metabolic pathways following databases were used: HMDB (http://www.hmdb.ca/), METLIN (http://metlin.scripps.edu/), Massbank (http://www.massbank.jp), and KEGG (http://www.kegg.com/).

Multivariate statistical analysis was performed using SIMCA-P software (version 14.1, Umetrics, Umea, Sweden). Unit variance scaling followed partial least-squares discrimination analysis (PCA) and orthogonal partial least-squares discriminant analysis (OPLS-DA), which were applied to distinguish three groups (viz., controls, idiopathic short stature group, and growth hormone deficiency group). A permutation test was used to check the validity and the degree of overfitting for the model. The VIP values of metabolites greater than 1 in non-targeted metabolomics analysis, and all metabolites were performed using the Wilcoxon Mann–Whitney test and identified various metabolites. *p* < 0.05 was considered significant. Meanwhile, the false discovery rate (FDR) was used for multiple comparisons (*p* < 0.10). The ratios of different metabolites between the average of those in normal control samples and the two experimental groups were calculated, and MeV version 4.5.1 software was used to illustrate the relationship between the different metabolites. Raw data are shown in Supplementary Table S1.

## Results

### Metabonomics Analysis in Two Different Types of Childhood Short Stature by UHPLC-TOF-MS

**FIGURE 1:**
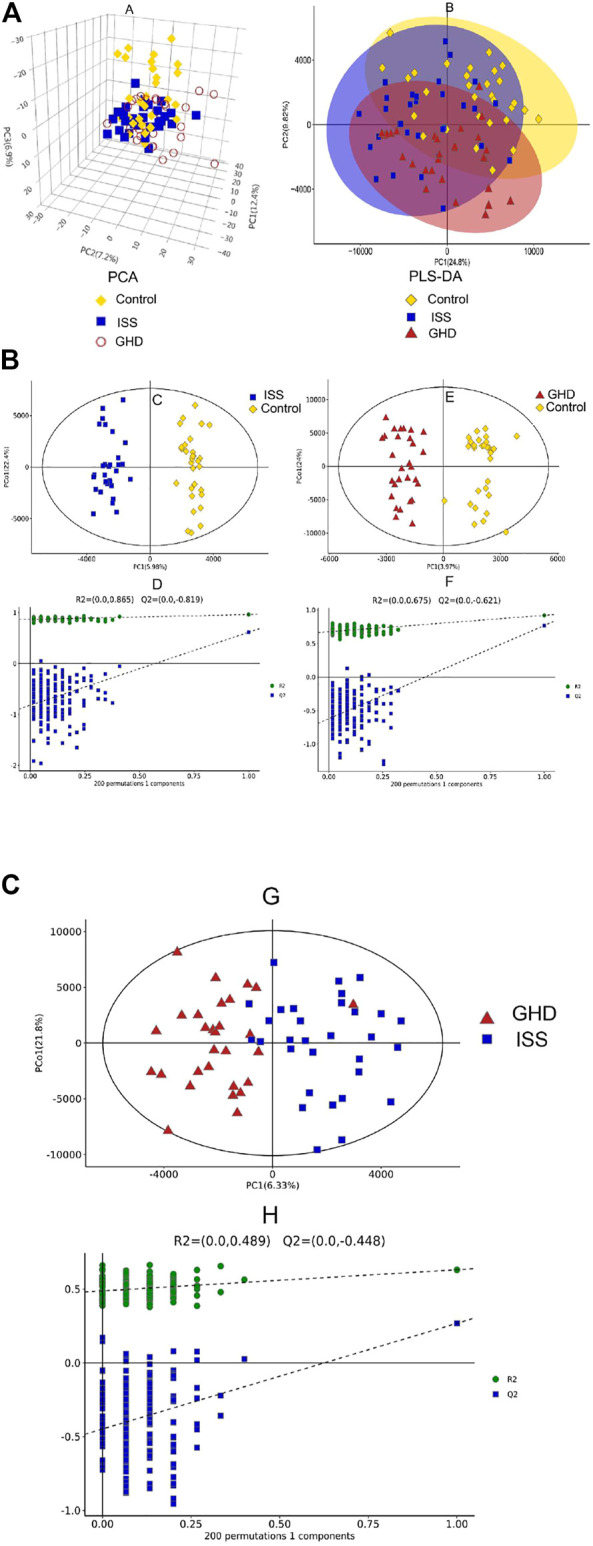
Metabolic profiling analysis in different types of childhood short stature. **(A)** Score plot of the samples using the 3D-PCA model. **(B)** Score plot of the samples using PLS-DA model. Control, normal height, *n* = 30; ISS, idiopathic short stature, *n* = 31; GHD, short stature of growth hormone deficiency, *n* = 29. **(C)** OPLS-DA between control group and ISS group. **(D)** Investigate the quality of the OPLS-DA model (control *vs*. ISS) by RPT (response permutation testing). **(E)** OPLS-DA between GHD group and control group. **(F)** Investigate the quality of the OPLS-DA model (GHD *vs*. control) by RPT (response permutation testing). Control, normal height, *n* = 30; ISS, idiopathic short stature, *n* = 31; GHD, short stature of growth hormone deficiency, *n* = 29. **(G)** OPLS-DA between ISS group and GHD group. **(H)** Investigate the quality of the OPLS-DA model (ISS *vs*. GHD) by RPT (response permutation testing). Control, normal height, *n* = 30; ISS, idiopathic short stature, *n* = 31; GHD, short stature of growth hormone deficiency, *n* = 29.

To explore the critical metabolites in serum metabolism of different types of childhood short stature, the metabolic profiles of serum in different groups (ISS and GHD groups) were analyzed by UHPLC-TOF-MS. Data were analyzed by SIMCA-P software. 3D PCA (principal components analysis) and PLS-DA (partial least-squares discrimination analysis) were demonstrated (Figure 1A). Orthogonal PLS-DA (OPLS-DA) was performed to explore further the risk metabolites in each group (Figure 1B). A clear differentiation was shown between the ISS and control groups, GHD and control groups, and ISS and GHD groups (Figure S1). All OPLS-DA models were reliable because none of the permutation tests had no overfitting. Many critical metabolites and multiple metabolic pathways had been explored in the serum of different types of childhood short stature.

### Visualization of Differential Metabolites Using Volcano Plot and Heat Map

**FIGURE 2:**
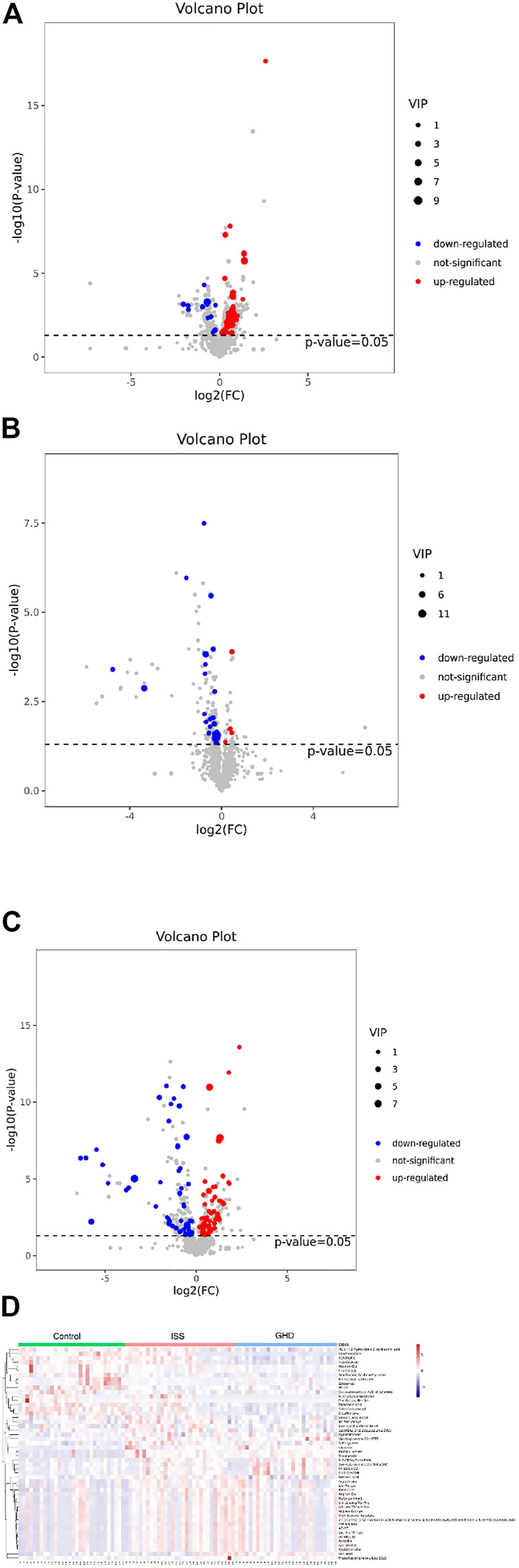
Differential metabolites demonstrated by volcano plot and heat map. **(A**–**C)** represent the metabolites that are downregulated, not significant, or upregulated in different types of childhood short stature. The abscissa is $\;log_{2}\left(FC\right)$ , the left ordinate is $-\log_{10}\left(p-value\right)$ , and the right ordinate is VIP (variable important in projection). **(A)** It presents volcano plot ISS *vs*. control. **(B)** It presents volcano plot ISS *vs*. GHD. **(C)** It presents volcano plot GHD *vs*. control. **(D)** It presents a heat map among ISS, GHD, and control. Control, normal height, *n* = 30; ISS, idiopathic short stature, *n* = 31, GHD, short stature of growth hormone deficiency, *n* = 29.

We investigated differential metabolites between the control group and two types of short stature group (Figures 2A–C). Compared with normal-height children, fourteen metabolites were downregulated, and 53 were upregulated in ISS (Figure 2A). Meanwhile, 50 metabolites were downregulated, and 63 were upregulated in the short stature of GHD (Figure 2C). Furthermore, we focused on 33 differential metabolites between the ISS and GHD groups (Figure 2B), including 27 downregulated and five upregulated metabolites. At the same time, the heat map revealed differential expression of metabolites among the control, ISS, and GHD groups. These findings revealed that specific metabolites played significant roles in the progression of different childhood SS.

### Metabolite–Metabolite Correlation Analysis Among Identified Metabolites

**FIGURE 3:**
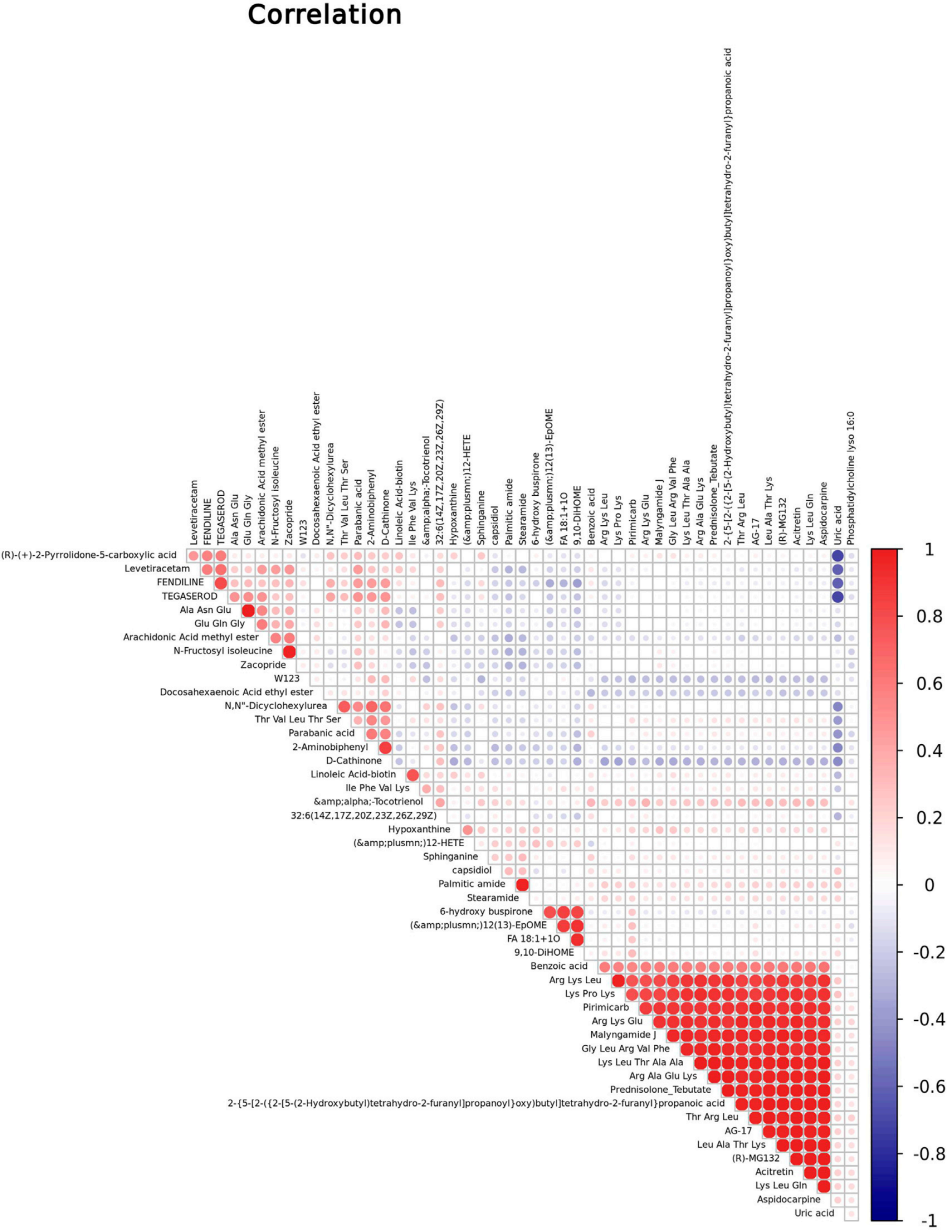
Correlation studies of identified metabolites in childhood short stature. Positive and negative correlations are indicated in red and blue, respectively.

We performed Pearson correlation coefficient analysis to understand further the interrelationship among identified metabolites in childhood short stature. Figure 3 shows the correlation between the top 50 differential metabolites. For instance, Thr Val Leu Thr Ser, one of the oligopeptides, shows a significant positive correlation with 2-aminobiphenyl and a negative correlation with uric acid.

### KEGG Pathway Enrichment Analysis

**FIGURE 4:**
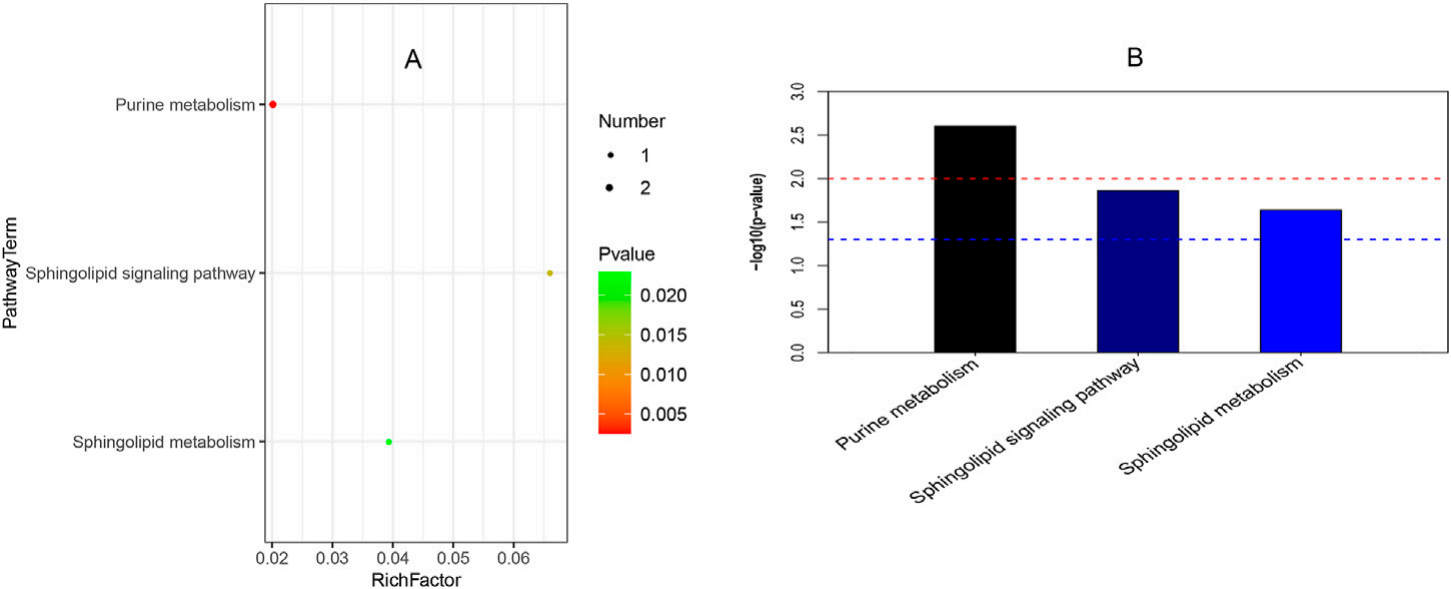
Pathway enrichment analysis. **(A)** It shows the bubble chart among ISS, GHD, and control groups. The abscissa is a rich factor (the detailed description was shown in Result 3.4), and the left ordinate is the pathway. **(B)** It presents the bar chart of the three groups. The abscissa is the pathway, and the ordinate is $-\log_{10}\left(p-value\right)$ . The blue line indicates a *p*-value threshold of 0.05, and the red line indicates a *p*-value threshold of 0.01.

Figure 4 reveals that three pathways (viz., purine metabolism, sphingolipid signaling pathway, and sphingolipid metabolism) were significantly enriched in childhood SS. In Figure 4A, the abscissa indicates rich factor originated in the number of differential metabolites in the corresponding metabolic pathway/the number of total metabolites identified in the pathway. The larger the value of the rich factor, the greater the degree of pathway enrichment. Colors are expressed on a linear scale from green to red with *p* values gradually decreasing. In addition, the greater the bubble, the more metabolites in the pathway. Bar charts in Figure 4B show purine metabolism was the most significantly enriched in the three groups.

### Short Peptides Analysis

**FIGURE 5:**
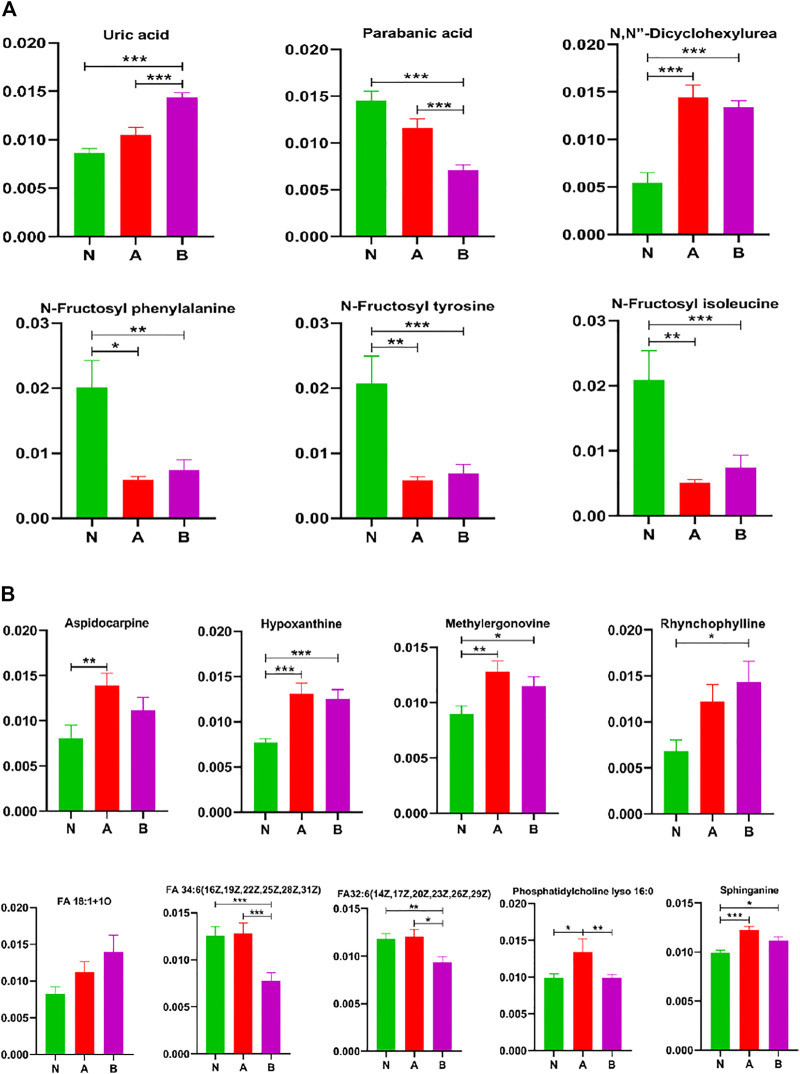
Other metabolites analysis in column configuration. Group N, normal height, *n* = 30; group A, ISS, idiopathic short stature, *n* = 31; group B, GHD, short stature of growth hormone deficiency, *n* = 29. All data are mean ± SEM. *p < 0.05; **p < 0.01; ***p < 0.001; two-tailed Mann–Whitney U test.

Short peptides include basic molecular information and are the precursor to life. Researchers have recently developed a greater interest in the field due to its unique features and rosy prospects in innovative bio-therapies [25]. There are 18 short peptides with visible differences between normal-height children (control) and idiopathic short stature (ISS) in Figure 5. They are Arg Ala Glu Lys, Arg Lys Glu, Arg Phe Val, Gly Leu Arg Val Phe, lle Phe Val Lys, Leu Ala Thr Lys, Lys Leu Gln, Lys Leu Thr Ala Ala, Lys Pro His, Lys Pro Lys, Lys Ser Gln Lys, Phe Ala Asn Lys, Ser Lys Phe, Thr Arg Leu, Thr Asn Phe Asp, Thr Glu Leu Lys, Trp Ile Lys, Val Ile Asp Lys. Similarly, 16 short peptides have shown a visible difference between normal-height children (control) and short stature caused by the GHD. They are Arg Ala Glu Lys, Arg Lys Leu, Arg Phe Val, Glu Gln Gly, Leu Ala Thr Lys, Lys Leu Gln, Lys Pro His, Lys Pro Lys, Lys Ser Gln Lys, Phe Ala Asn Lys, Ser Lys Phe, Thr Asn Phe Asp, Thr Glu Leu Lys, Thr Val Leu Thr Ser, Trp Ile Lys, and Val Ile Asp Lys. Furthermore, there are merely five short peptides visibly different in ISS and GHD short stature children. They are Arg Lys Leu, Glu Gln Gly, lle Phe Val Lys, Thr Val Leu Thr Ser, and Trp Ile Lys.

We found that the short peptides Thr Val Leu Thr Ser and Trp Ile Lys might significantly affect childhood short stature.

### 3.6 Arachidonic Acid Pathway Analysis

**FIGURE 6:**
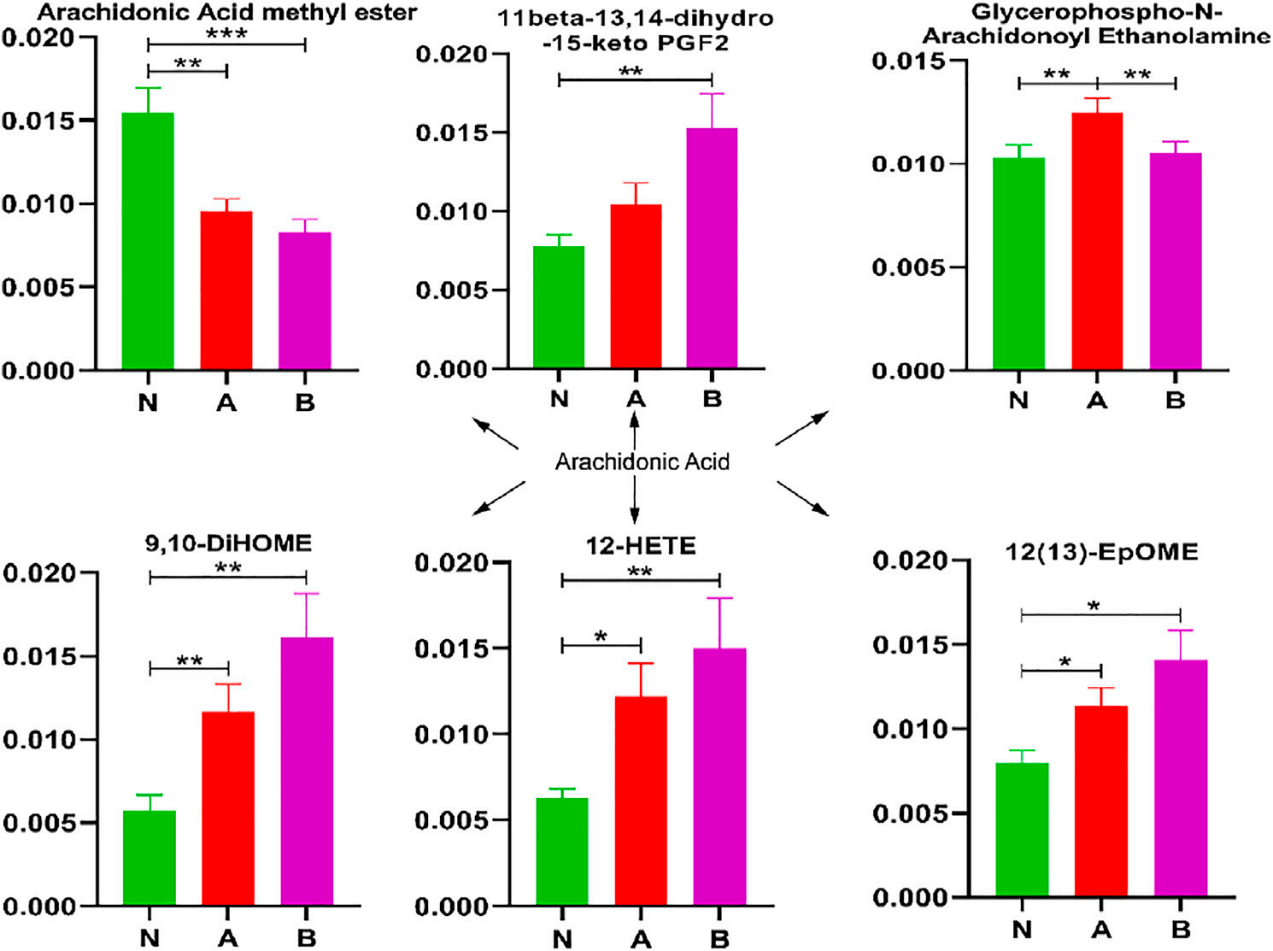
Arachidonic acid pathway analysis in column configuration. Group N, normal height, *n* = 30; group A, ISS, idiopathic short stature, *n* = 31; group B, GHD, the short stature due to growth hormone deficiency, *n* = 29. All data are mean ± SEM. *p < 0.05; **p < 0.01; ***p < 0.001; two-tailed Mann–Whitney U test.

In children with idiopathic short stature or a growth hormone deficiency, 9,10-DiHOME, 12-HETE, and 12 (13)-EpOME significantly increased, whereas arachidonic acid methyl ester was remarkably decreased (Figure 6). Glycerophospho-N-arachidonoyl ethanolamine showed markedly elevated levels in children with idiopathic short stature compared to normal-height children or children with growth hormone deficiency. Thus, these metabolites refined our understanding of SS in the arachidonic acid pathway.

### Acids and Acid Amides Metabolites Analysis

**FIGURE 7:**
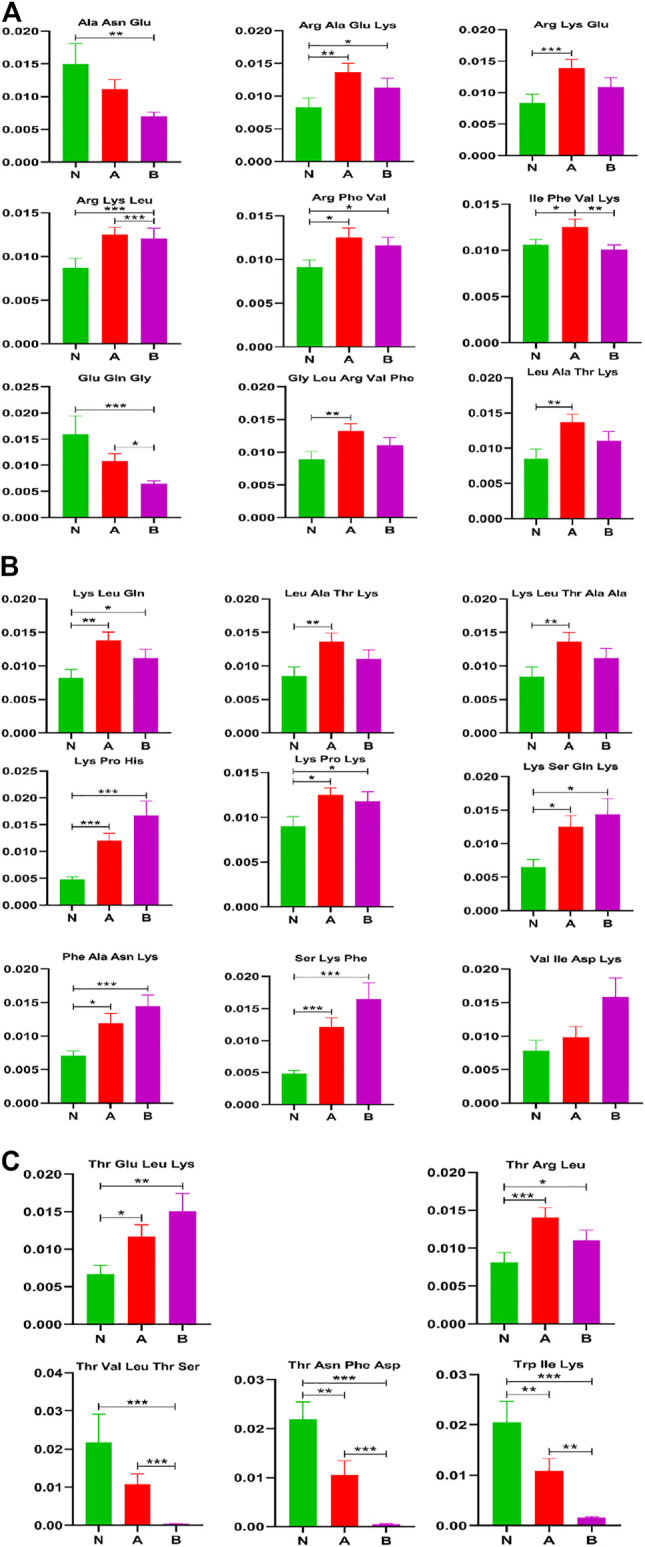
Acids and acid amides metabolite(s) analysis in column configuration. Group N, normal height, *n* = 30; group A, ISS, idiopathic short stature, *n* = 31; group B, GHD, short stature of growth hormone deficiency, *n* = 29. All data are mean ± SEM. *p < 0.05; **p < 0.01; ***p < 0.001; two-tailed Mann–Whitney U test.

To better understand differential metabolites in pubertal children with short stature, acids and acid amides were also analyzed to find the key metabolites that could differentiate between the normal-height group and SS (Figure 7). Our results demonstrated that the key metabolites were curvulinic acid, docosahexaenoic acid ethyl ester, nonanoic acid, stearamide, capsidiol, eicosanoyl-EA, N'-(2,4-dimethylphenyl)-N-methylformamidine, N, N-diisopropyl-3-nitrobenzamide, and palmitic amide. Moreover, the compounds such as alpha-tocotrienol, benzoic acid, curvulinic acid, linoleic acid-biotin, nonanoic acid, and N'-(2,4-dimethylphenyl)-N-methylformamidine could discriminate ISS group from normal height subjects or children with the GHD.

It was observed that some metabolites could distinguish subtypes of childhood SS, which were (R)-(+)-2-pyrrolidone-5-carboxylic acid, alpha-tocotrienol, benzoic acid, curvulinic acid, linoleic acid-biotin, nonanoic acid, and N'-(2,4-dimethylphenyl)-N-methylformamidine.

### Analysis of Other Metabolites

**FIGURE 8:**
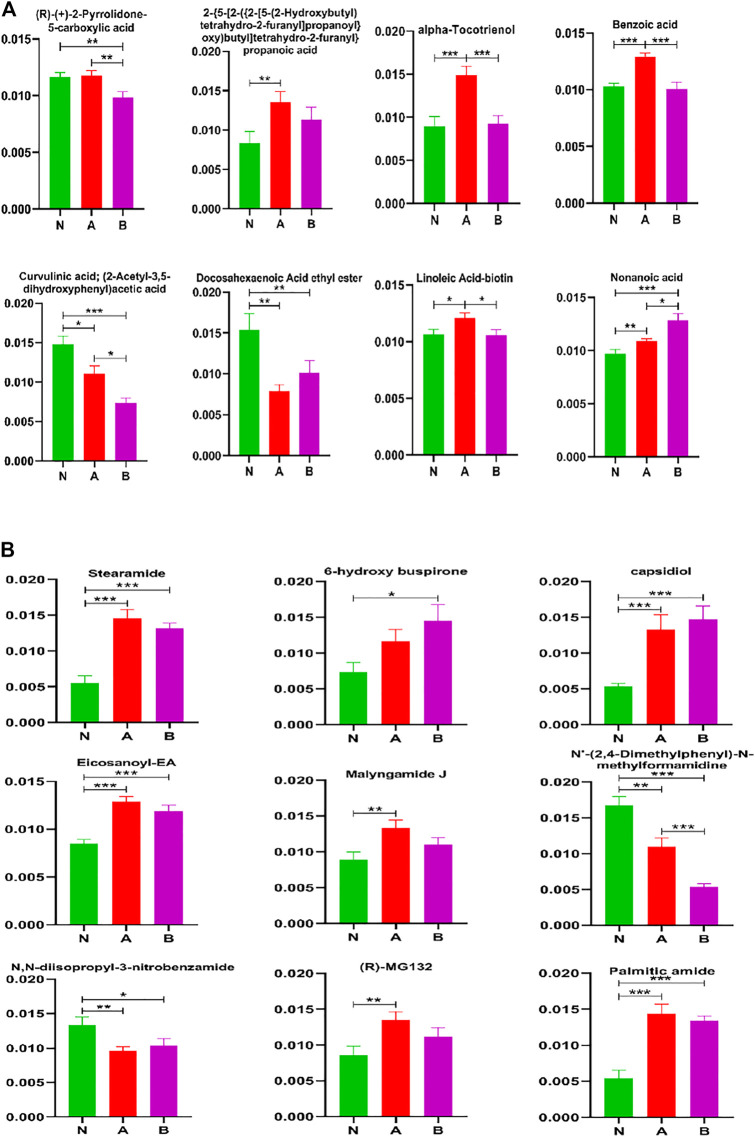
Other metabolites analysis in column configuration. Group N, normal height, *n* = 30; group A, ISS, idiopathic short stature, *n* = 31; group B, GHD, short stature of growth hormone deficiency, *n* = 29. All data are mean ± SEM. *p < 0.05; **p < 0.01; ***p < 0.001; two-tailed Mann–Whitney U test.

While screening differential metabolites, we investigated some interesting compounds like uric acid, lipids, fructose, and others in (Figure 8). A series of metabolites (Yusupov et al., 2017) could differentiate between the normal-height group and childhood SS, that is, N, N-dicyclohexylurea, N-fructosyl isoleucine, N-fructosyl phenylalanine, N-fructosyl tyrosine, methylergonovine, hypoxanthine, and sphinganine. Moreover, phosphatidylcholine lyso 16:0 could discriminate ISS from normal-height subjects or children with growth hormone deficiency. We also discovered some metabolites could identify subtypes of childhood SS, which were parabanic acid, FA 34:6 (16Z,19Z,22Z,25Z,28Z,31Z), and FA32:6 (14Z,17Z,20Z,23Z,26Z,29Z).

## Discussion

The most significant difference between the species is the size of the individual (Klingseisen and Jackson, 2011). However, the mechanisms behind childhood short stature’s pathogenic processes remain poorly understood. Early diagnosis and identification of key metabolites of childhood SS will lead to the reasonable treatment and understanding of the mechanism behind the progression of the disease. Our study investigates the key metabolites between different types of childhood SS and normal height children by UHPLC-MS-MS.

Our study revealed that purine metabolism, sphingolipid signaling pathway, and sphingolipid metabolism were significantly enriched in ISS, childhood SS with GHD, and normal-height children (Figure 4). Several studies involving the anti-aging gene SIRT1 have suggested that SIRT1 is involved in regulating growth hormone and sphingolipid metabolism with relevance in neurodegeneration. Therefore, it has been suggested that SIRT1 plays a critical role in the hypothalamic–pituitary axis, regulation of metabolism, aging, and longevity (Satoh et al., 2011; Yamamoto and Takahashi, 2018).

Some studies have observed that purine metabolism plays a vital role in energy metabolism (Esther et al., 2015). Since uric acid is the final product and an essential index of purine metabolism (Si et al., 2020), we found that increased uric acid could discriminate idiopathic short stature among the ISS, GHD, and control groups (Figure 8). Moreover, hypoxanthine could distinguish childhood SS from normal height children, as observed in Figure 8. Changed sphingolipid metabolism might result in sensory neuron damage (Chen et al., 2015). In recent years, some evidence has also suggested that sphingolipid dysregulation plays a pivotal role in the pathogenesis of many brain disorders (Bourgognon et al., 2018). Growth hormone secreted from the pituitary gland is closely related to childhood SS. The primary reasons for GH secretion level changes with age were associated with the transformation in the pituitary gene expression of GH and the GHRH receptor together with a decrease in hypothalamic GHRH (Sonntag et al., 1980; Corpas et al., 1993; Chapman et al., 1997; Degli Uberti et al., 1997; Russell-Aulet et al., 1999; Rosen, 2000; Frutos et al., 2007; Weigent, 2013). Our results showed that sphinganine could distinguish between childhood SS and normal height children. In addition, sphingolipid metabolites have also been shown to regulate arachidonic acid (AA) metabolism (Zhang et al., 2021). As shown in Figure 6, similarly, our results have revealed that the AA pathway contributes to childhood SS development and progression.

Short peptides play an important role in the immune system and are responsible for transmitting most immunological information (Parhiz et al., 2013). Some short peptides (2–20 amino acids) are partially obtained by enzymatic hydrolysis of proteins and are important bioactive peptides (Zhang et al., 2019). In recent years, short peptides have been of considerable interest in the branch of biology, chemistry, and medicine for their unique structural and functional diversity (Apostolopoulos et al., 2021). Reports showed that short peptides have relevance in many diseases, including neurodegenerative diseases (Jang et al., 2016), Alzheimer’s (Perez-Garmendia and Gevorkian, 2013; Soudy et al., 2019), and rheumatoid arthritis (Turesson et al., 2007; Ruiz-Ortiz De Arrizabaleta et al., 2011) and also has many therapeutic effects (New et al., 2014; Tatman et al., 2016; Baig et al., 2018; Horsley et al., 2020). Our results demonstrated that 18 short peptides could visibly differentiate between the control and ISS groups, as well as 16 short peptides could clearly distinguish the control from the GHD group, and six short peptides can distinguish ISS from the GHD group (Figure 5). These findings necessitate further investigation and may serve as the starting point for developing new therapeutics for childhood SS.

Our study also detected a number of acids, acid amides, lipids, fructose, and other metabolites, indicating their involvement in the process of childhood SS (Figures 6-8). The 16 metabolites that could differentiate between the normal height group and childhood SS were curvulinic acid, docosahexaenoic acid ethyl ester, nonanoic acid, stearamide, capsidiol, eicosanoyl-EA, N'-(2,4-dimethylphenyl)-N-methylformamidine, N,N-diisopropyl-3-nitrobenzamide, palmitic amide, N,N-dicyclohexylurea, N-fructosyl isoleucine, N-fructosyl phenylalanine, N-fructosyl tyrosine, methylergonovine, hypoxanthine, and sphinganine. Furthermore, the seven compounds that could discriminate ISS from normal-height subjects or childhood SS with GHD were alpha-tocotrienol, benzoic acid, curvulinic acid, linoleic acid-biotin, nonanoic acid, N'-(2,4-dimethylphenyl)-N-methylformamidine, and phosphatidylcholine lyso 16:0.

Our study has also revealed that 10 metabolites could distinguish subtypes of childhood SS, which were (R)-(+)-2-pyrrolidone-5-carboxylic acid, alpha-tocotrienol, benzoic acid, curvulinic acid, linoleic acid-biotin, nonanoic acid, parabanic acid, FA 34:6 (16Z,19Z,22Z,25Z,28Z,31Z) FA32:6 (14Z,17Z,20Z,23Z,26Z,29Z), and N'-(2,4-dimethylphenyl)-N-methylformamidine. However, in order to achieve a clear distinction and characterization, optimization of potential biomarker analysis is the most important to understand childhood SS. In our study, the three potential biomarkers, curvulinic acid (2-acetyl-3,5-dihydroxyphenyl acetic acid), nonanoic acid, and N'-(2,4-dimethylphenyl)-N-methylformamidine had apparent changes among the three groups. It has been observed that curvulinic acid originated from the microbial metabolism of polyphenols is widely distributed in organisms (Vázquez-Manjarrez et al., 2020). Polyphenols have been shown to play an essential part in plant development (participating in plant hormone signaling), reproduction, and defense (protecting from pathogens) (Agati et al., 2012; Agati et al., 2013; Zhao et al., 2017). Nonanoic acid is a species of fatty acid and is a group of compounds that can potentially be a consequence of increased cell membrane lysis (Hillyer et al., 2016; Yusupov et al., 2017; Lawson et al., 2019). Some studies have also demonstrated that nonanoic acid had a particular influence on the Poria placenta growth (Kozicki et al., 2019). Some genes can degrade an array of methylated compounds (dimethylamine, methylamine, and N'-(2,4-dimethylphenyl)-N-methylformamidine) to produce either formaldehyde or methyl groups as the intermediates (Huang et al., 2019). Consequently, our study revealed that these three potential biomarkers played vital roles in the development of childhood SS.

There are some limitations of this study that need to be focused and discussed. Even though there were relatively equal numbers of gender and age in each childhood SS group, we were underpowered to evaluate the associated diseases (except for injuries), dietary supplements, and diet differences. Furthermore, the batch samples used for screening key metabolites were relatively modest (normal height, *n* = 30; ISS, *n* = 31; GHD, *n* = 29), although the number of samples met the human metabonomics requirements. Nevertheless, our observations need to be replicated in an independent and enough quantity samples in the future. Moreover, childhood SS could be primarily judged by body height comparison table and instruments (test bone age). We noted that our study was intended to illustrate complementary methods that might improve clinical prediction and help understand childhood SS progression.

## Conclusion

**FIGURE 9:**
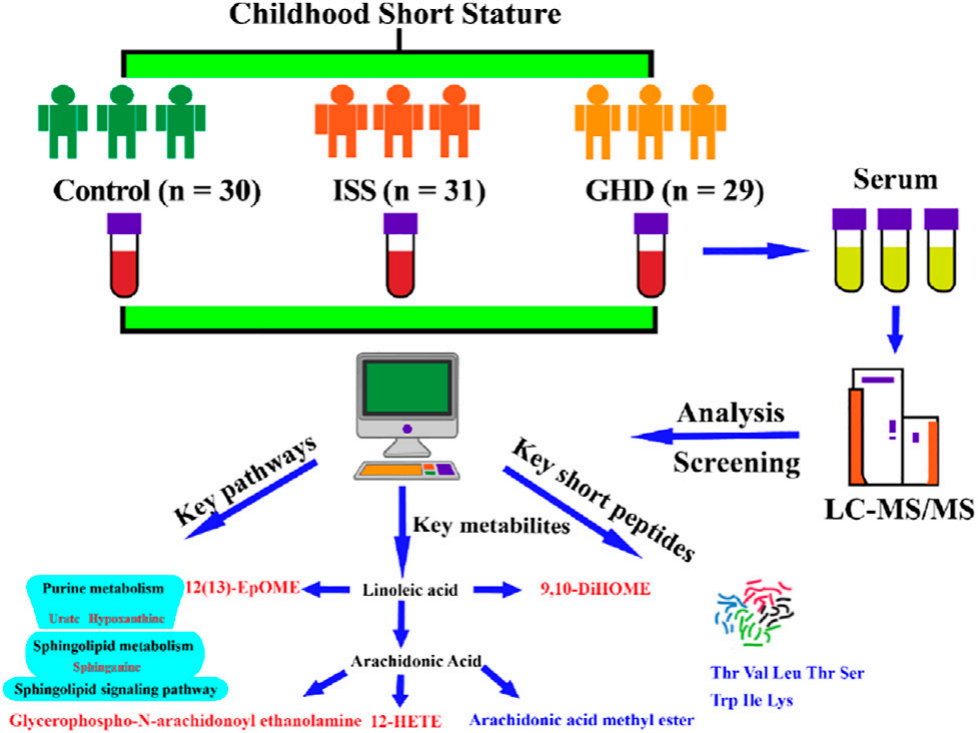
Control, normal height, *n* = 30; ISS, idiopathic short stature, *n* = 31; GHD, short stature of growth hormone deficiency, *n* = 29. Significance was increased relative to the controls indicated in red, and significance was decreased relative to the controls indicated in blue. All data are of the two-tailed Mann–Whitney U test.

In our study, the metabolite changes in ISS and short stature caused by GHD were investigated utilizing UHPLC-Q-TOF-MS-based metabonomics (Figure 9). Our data have revealed that three pathways (viz., purine metabolism, sphingolipid signaling pathway, and sphingolipid metabolism) were significantly enriched in childhood SS. Moreover, two short peptides (Thr Val Leu Thr Ser and Trp Ile Lys) may play a vital role in childhood SS. In addition, altered metabolites were involved in the metabolism of 9,10-DiHOME, 12-HETE, and 12 (13)-EpOME, arachidonic acid methyl ester, glycerophospho-N-arachidonoyl ethanolamine, curvulinic acid (2-acetyl-3,5-dihydroxyphenyl acetic acid), nonanoic acid, and N'-(2,4-dimethylphenyl)-N-methylformamidine. Our findings contribute to the understanding of molecular metabolic processes in childhood short stature and may provide potential clues for the underlying mechanisms that will help treat childhood short stature.

## Data Availability

The original contributions presented in the study are included in the article/Supplementary Material; further inquiries can be directed to the corresponding authors.
